# Effect of Empagliflozin on Cardiac Function, Adiposity, and Diffuse Fibrosis in Patients with Type 2 Diabetes Mellitus

**DOI:** 10.1038/s41598-019-51949-5

**Published:** 2019-10-25

**Authors:** Jung-Chi Hsu, Chih-Yuan Wang, Mao-Yuan M. Su, Lian-Yu Lin, Wei-Shiung Yang

**Affiliations:** 1 Department of Internal Medicine, Division of Cardiology, Saint Mary’s Hospital Luodong, Yilan, 26546 Taiwan; 2 Graduate Institute of Clinical Medicine, National Taiwan University, Taipei, 10617 Taiwan; 3 Department of Internal Medicine, Division of Endocrinology, National Taiwan University Hospital and College of Medicine, National Taiwan University, Taipei, 10617 Taiwan; 4 Department of Medical Imaging, National Taiwan University of College of Medicine, Taipei, 10617 Taiwan; 5 Department of Internal Medicine, Division of Cardiology, National Taiwan University Hospital and College of Medicine, National Taiwan University, Taipei, 10617 Taiwan

**Keywords:** Cardiomyopathies, Type 2 diabetes

## Abstract

Empagliflozin, a sodium-glucose cotransporter 2 (SGLT2) inhibitor, significantly improves cardiovascular outcomes in diabetic patients; however, the mechanism is unclear. We hypothesized that empagliflozin might have beneficial effects on cardiac function, structure, adiposity, and myocardial diffuse fibrosis. This prospective study enrolled 35 patients (48.6% men, age 63.5 ± 9.7 years) with type 2 diabetes mellitus (T2DM) from June 1, 2017, to November 31, 2018. The patients received an SGLT2 inhibitor (empagliflozin 25 or 12.5 mg/d) for 6 months in addition to stable oral hypoglycaemic treatment. All patients underwent cardiac magnetic resonance imaging (CMRI) before and after empagliflozin treatment. Left ventricular (LV) function and structure were quantified using cine CMRI. Cardiac adiposity was defined based on pericardial fat and intracardiac triglyceride contents, whereas myocardial diffuse fibrosis was indicated by extracellular volume (ECV). The statistical significance of parameter changes was assessed using paired t-test and stepwise multiple linear regression. There were no significant differences in LV function and structure changes. Cardiac adiposity and diffuse fibrosis indices were also not different before and after empagliflozin treatment. Concerning clinical parameters, only a significant decrease in systolic blood pressure (by 6.4 mmHg) was observed (p = 0.013). Stepwise multiple linear regression revealed that worse baseline MRI parameters were associated with better improvements. Intracardiac triglyceride content decrease was inversely associated with baseline intracardiac triglyceride content (p < 0.001). Pericardial fat changes were negatively correlated with baseline pericardial fat (p < 0.001) and ECV changes (p = 0.028). ECV changes were inversely associated with baseline ECV (p < 0.001), baseline LV ejection fraction (p < 0.001), and LV mass index changes (p = 0.020). This study demonstrated that 6 months of empagliflozin treatment did not significantly improve the LV function, structure, adiposity, and diffuse fibrosis in patients with T2DM. Further, the beneficial effects of empagliflozin treatment might be more evident in patients with worse baseline LV substrate and structure.

## Introduction

Empagliflozin, a sodium-glucose cotransporter 2 (SGLT2) inhibitor, represents a new milestone in the treatment of type 2 diabetes mellitus (T2DM). The EMPA-REG OUTCOME trial showed that empagliflozin significantly reduced the heart failure hospitalization rate and lowered the cardiac mortality rate in patients with T2DM with a high cardiovascular (CV) risk^1^. However, the underlying mechanisms leading to these beneficial effects remain not well elucidated. As the human myocardium does not express SGLT2 receptors, many researchers have been attempting to find the link between pharmacological mechanisms and favourable outcomes.

A potential explanation for the beneficial effects is better glycaemic control. However, a systematic review showed no difference in haemoglobin A1c (HbA1c) levels among SGLT2 inhibitors and other oral pharmacological treatment of T2DM^2^. In addition, the benefits of empagliflozin on heart failure hospitalization and CV death were evident despite the magnitude of HbA1c reduction or the baseline HbA1c^3^. These findings suggest that the effect of empagliflozin is independent of glycaemia control. Empagliflozin significantly lowers the systolic and diastolic blood pressure, which contributes to the reduction of heart loading. However, the fact that the incidence of stroke did not decrease in the EMPA-REG OUTCOME trial casts doubt on the osmotic diuretic effect as the only explanation^4^. Empagliflozin treatment also changes the fuel and enhances the energy status of the heart by increasing ATP production^5^. An experimental model demonstrated that empagliflozin could increase the rate of glucose and fatty acid oxidation, rather than ketone oxidation, in diabetic mice, leading to an increase in the circulating ketone levels and thus providing the failing heart with additional source of energy^6^. The study has set a new translational horizon; however, it focused more on systolic rather than diastolic function. Further, as glucagon increases the heart rate, the theory of shifting fuel does not fully support the results of a relative stable heart rate in the EMPA-REG OUTCOME trial^7^. Recently, one study showed that treatment with empagliflozin for 3 months significantly reduced the left ventricular (LV) mass index (LVMi) and improved diastolic function^8^. This study indicated that the beneficial effects of empagliflozin might be attributed to LV reverse remodelling.

Cardiac magnetic resonance imaging (CMRI) is a promising tool for evaluating the structure, function, and myocardial contents of the left ventricle. With T1 mapping, CMRI could measure the extracellular volume (ECV) of the myocardium, which is an indicator of diffuse myocardial fibrosis. Diffuse myocardial fibrosis is the core of LV remodelling and plays a unique role in the pathogenesis of heart failure with preserved ejection fraction^9–11^. CMRI could also quantify the cardiac adiposity by measuring the pericardial fat volume and the intramyocardial triglyceride (TG) content (cardiac steatosis). It is well known that pericardial fat and cardiac steatosis are associated with LV diastolic dysfunction and might contribute to the generation and progression of diffuse myocardium fibrosis^12–14^.

The current study was designed to investigate whether empagliflozin treatment for 6 months could have beneficial effects on the cardiac function, structure, and myocardial contents of the left ventricle. The aim of this study was to analyse the changes of CMRI parameters before and after empagliflozin treatment, and to identify the predictors leading to favourable LV remodelling.

## Results

### Baseline characteristics

A total of 35 patients were included. The average age was 63.5 ± 9.7 years, and 17 (48%) patients were men. The mean systolic/diastolic blood pressure was 132.3 ± 17.4/75.5 ± 12.6 mmHg. Most patients had hypertension (82.9%) and dyslipidaemia (74.3%), and about half of the patients had a history of coronary artery disease (CAD; 48.6%). All patients were enrolled from the outpatient department and had no signs of acute decompensated heart failure. The mean serum N-terminal-pro-brain natriuretic peptide (NT-proBNP) level was 64.32 ± 66.58 pg/mL. The mean HbA1c was 7.0 ± 1.1%. Beta-blocker, angiotensin-converting enzyme inhibitor, and angiotensin II receptor blocker were administered in 45.7%, 22.9%, and 48.6% of the patients, respectively. With respect to T2DM medications, 74.3% received metformin, 14.3% received sulfonylurea, 2.9% received glinide, 31.1% received pioglitazone, and 5.7% of the patients received insulin treatment. As the national health insurance does not allow the combination use of dipeptidyl-peptidase-4 inhibitor and SGLT2, no patient received this combination treatment during the study. There was only one patient with impaired systolic function, with a mean LV ejection fraction (LVEF) of 70.1 ± 8.0%. The amount of intracardiac TG content, pericardial fat, ECV, and other CMRI parameters are listed in Table 1.

**Table 1:** Patients’ baseline characteristics.

Patients (N = 35)	Number (%) or mean ± SD
Sex (male)	17 (48.6%)
Age (y)	63.5 ± 9.7 (43–80)
BW (kg)	70.4 ± 14.5 (49.0–107.0)
BMI (kg/m^2^)	26.6 ± 3.9 (19.8–37.8)
SBP (mmHg)	132.3 ± 17.4 (88–162)
DBP (mmHg)	75.5 ± 12.6 (44–109)
**Patient history**	
Smoking	4 (11.4%)
Alcohol	1 (2.9%)
Hypertension	29 (82.9%)
Dyslipidaemia	26 (74.3%)
Gout	2 (5.7%)
Previous myocardial infarction	6 (17.1%)
Coronary artery disease	17 (48.6%)
Previous cerebrovascular accident	1 (2.9%)
Chronic kidney disease	3 (8.6%)
**Laboratory data**	
Haematocrit (%)	41.8 ± 4.5 (30.3–54.5)
NT-proBNP (pg/mL)	64.32 ± 66.58 (8.84–283.30)
Creatinine (mg/dL)	0.9 ± 0.3 (0.5–1.6)
eGFR (mL/min/1.73 m^2^)	82.3 ± 19.4 (46.3–132.9)
HbA1c (%)	7.0 ± 1.1 (5.3–11.7)
T-CHO (mg/dL)	167.5 ± 39.3 (68.0–237.0)
TG (mg/dL)	160.1 ± 94.1 (51.0–401.0)
LDL (mg/dL)	88.1 ± 22.5 (48.0–144.0)
HDL (mg/dL)	50.3 ± 11.8 (28.0–77.0)
**Medication**	
Aspirin	14 (40%)
Clopidogrel	1 (2.9%)
Beta-blocker	16 (45.7%)
Non-DHP CCB	3 (8.6%)
DHP CCB	11 (31.4%)
ACE inhibitor	8 (22.9%)
ARB	17 (48.6%)
Furosemide	2 (5.7%)
Spironolactone	1 (2.9%)
Alpha-blocker	12 (34.3%)
Nitrate	2 (5.7%)
Metformin	26 (74.3%)
Sulfonylurea	5 (14.3%)
Glinide	1 (2.9%)
Pioglitazone	13 (31.1%)
Insulin	2 (5.7%)
Statin	28 (80%)
**CMRI parameters**	
Myocardial TG (%)	0.048 ± 0.340 (0.013–1.690)
Pericardial fat (g)	32.26 ± 14.60 (5.74–82.76)
ECV (%)	27.40 ± 4.08 (22.42–42.65)
LVEDV (mL)	94.38 ± 28.21 (41.00–162.00)
LVESV (mL)	24.54 ± 19.56 (2.54–79.86)
nLVEDV (mL/m^2^)	53.46 ± 16.20 (25.56–100.11)
nLVESV (mL/m^2^)	13.81 ± 11.94 (1.57–56.78)
LVEF (%)	77.23 ± 12.12 (43.28–93.86)
LVPER (ESV/s)	−3.74 ± 1.01 (−5.62 to −1.41)
LVPFR (EDV/s)	3.87 ± 1.55 (0.57–6.38)
LVMi (g/m^2^)	95.11 ± 28.26 (35.39–151.23)

SD: standard deviation; CMRI: cardiac magnetic resonance imaging; BW: body weight; BMI: body mass index; SBP: systolic blood pressure; DBP: diastolic blood pressure; NT-proBNP: N-terminal pro b-type natriuretic peptide; eGFR: estimated glomerular filtration rate; T-CHO: total cholesterol; TG: triglyceride; LDL: low-density lipoprotein; HDL: high-density lipoprotein DHP CCB: dihydropyridine calcium channel blocker; ACE: angiotensin-converting enzyme; ARB: angiotensin II receptor blocker; ECV: extracellular volume; LVEDV: left ventricular end-diastolic volume; LVESV: left ventricular end-systolic volume; nLVEDV: normalized LVEDV (by body surface area); nLVESV: normalized LVESV (by body surface area); LVEF: left ventricle ejection fraction; LVPER: left ventricular peak ejection rate; LVPFR: left ventricular peak filling rate; LVMi: left ventricular mass index.

### Changes of LV structure, function, and substrates before and after empagliflozin treatment

**Figure 1:**
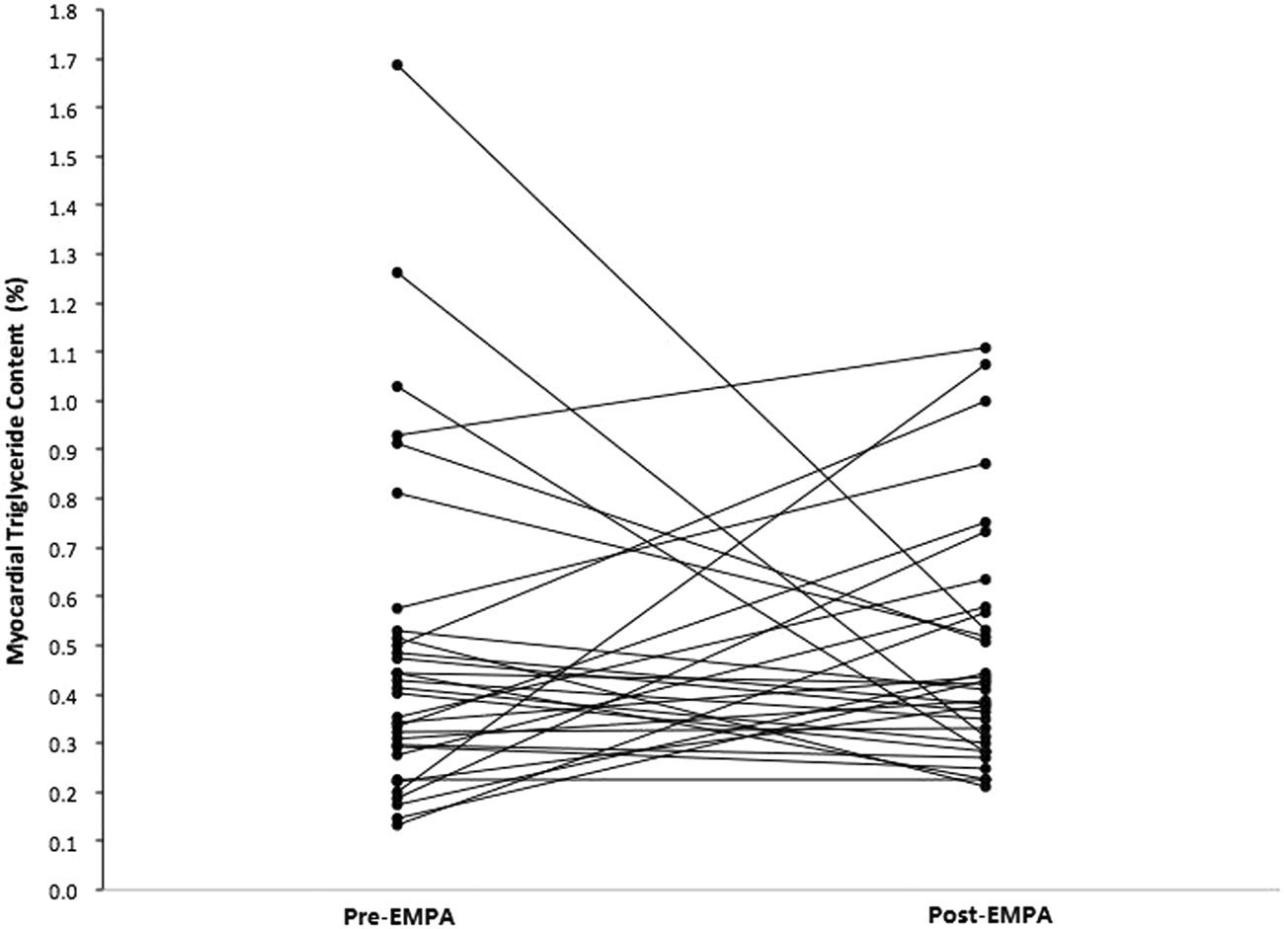
Myocardial triglyceride before and after empagliflozin (EMPA) treatment.

**Figure 2:**
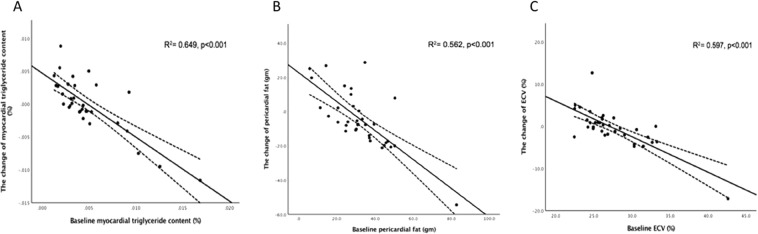
Correlations between (**A**) baseline intracardiac triglyceride and its change, (**B**) baseline pericardial fat and its change, and (**C**) baseline extracellular volume (ECV) and its change.

The CMRI parameters of the patients are summarized in Table 2. There were no differences in intracardiac TG, pericardial fat, ECV, normalized LV end-diastolic volume (nLVEDV), normalized LV end-systolic volume (nLVESV), LVEF, LV peak ejection rate (LVPER), LV peak filling rate (LVPFR), and LVMi before and after empagliflozin treatment. A significant systolic blood pressure decrease of 6.4 mmHg was observed (p = 0.013). The blood haematocrit level was significantly increased (p = 0.001). Body weight, NT-proBNP, HbA1c, and lipid profile did not show significant differences. The change of intracardiac TG content after empagliflozin is demonstrated in Fig. 1. The results of multiple linear regression analyses are listed in Table 3. The decrease of intracardiac TG content was inversely associated with the baseline intracardiac TG content (p < 0.001). The change of pericardial fat was negatively correlated with the baseline pericardial fat (p < 0.001) and the change of ECV (p = 0.028). The change of ECV was inversely associated with baseline ECV (p < 0.001), baseline LVEF (p < 0.001), and change of LVMi (p = 0.020). The correlation of intracardiac TG content, pericardial fat, and ECV and their baseline characteristics are shown in Fig. 2.

**Table 2:** Paired sample t-test comparing pre-empagliflozin and post-empagliflozin variables.

CMRI parameters	Pre-empagliflozin	Post-empagliflozin	Paired difference		95% confidence of the difference		p-Value
	Mean ± SD	Mean ± SD	Mean	SD	Lower	Upper	
Intracardiac TG (%)	0.478 ± 0.340 (0.013–1.690)	0.480 ± 0.246 (0.210–1.110)	0.002	0.418	−0.148	0.153	0.973
Pericardial fat (g)	32.26 ± 14.60 (5.74–82.76)	27.68 ± 11.12 (12.89–63.34)	4.576	16.432	−1.069	10.221	0.109
ECV (%)	27.4 ± 4.08 (22.42–42.65)	27.1 ± 2.89 (19.90–37.42)	0.299	4.439	−1.225	1.824	0.692
LVEDV (mL)	94.38 ± 28.21 (41.00–162.00)	95.00 ± 24.66 (50.33–140.8)	−0.614	17.795	−6.727	5.499	0.839
LVESV (mL)	24.54 ± 19.56 (2.54–79.86)	25.43 ± 17.11 (4.30–77.55)	−0.820	12.424	−5.164	3.372	0.672
nLVEDV (mL/m^2^)	53.46 ± 16.20 (25.56–100.11)	57.80 ± 18.60 (34.95–129.91)	−4.344	19.769	−11.135	2.447	0.202
nLVESV (mL/m^2^)	13.81 ± 11.94 (1.57–56.78)	15.17 ± 10.77 (4.60–49.23)	−1.354	6.169	−3.473	0.765	0.203
LVEF (%)	77.23 ± 12.12 (43.28–93.86)	75.19 ± 11.20 (41.48–91.46)	2.035	6.610	−0.235	4.306	0.077
LVPER (ESV/s)	−3.74 ± 1.01 (−5.62 to −1.41)	−3.72 ± 1.24 (−5.93 to −2.31)	−0.015	0.841	−0.304	0.274	0.919
LVPFR (EDV/s)	3.87 ± 1.55 (0.57–6.38)	3.73 ± 1.24 (1.91–6.17)	0.136	1.064	−0.229	0.502	0.454
LVMi (g/m^2^)	95.11 ± 28.26 (35.39–151.23)	91.81 ± 26.67 (46.52–135.26)	3.302	13.932	−1.484	8.088	0.170
**Others**							
BW (kg)	70.4 ± 14.5 (49.0–107.0)	69.9 ± 15.0 (49.0–110.0)	0.647	1.631	0.078	1.216	0.109
SBP (mmHg)	132.3 ± 17.4 (88–162)	125.9 ± 15.1 (95–162)	6.371	14.390	1.428	11.314	0.013
DBP (mmHg)	75.5 ± 12.6 (44–109)	75.2 ± 9.3 (56–99)	0.371	11.188	−3.472	4.325	0.845
Haematocrit (%)	41.8 ± 4.5 (30.3–54.5)	43.1 ± 4.2 (35.8–53.8)	1.400	2.200	0.600	2.200	0.001
NT-pro-BNP (pg/mL)	64.32 ± 66.58 (8.84–283.30)	67.62 ± 71.33 (7.35–379.80)	0.691	51.583	−18.230	19.612	0.942
Creatinine (mg/dL)	0.9 ± 0.3 (0.5–1.6)	0.9 ± 0.3 (0.6–1.7)	−0.147	0.089	−0.046	0.016	0.343
eGFR (mL/min/1.73 m^2^)	82.3 ± 19.4 (46.3–132.9)	80.1 ± 18.5 (41.6–116.0)	2.156	10.362	−1.460	5.771	0.234
HbA1c (%)	7.0 ± 1.1 (5.3–11.7)	6.8 ± 0.6 (5.8–8.3)	0.200	0.903	−0.220	0.520	0.199
T-CHO (mg/dL)	167.5 ± 39.3 (68.0–237.0)	160.0 ± 28.7 (105.0–239.0)	11.554	47.595	−7.670	30.778	0.227
TG (mg/dL)	160.1 ± 94.1 (51.0–401.0)	144.9 ± 70.4 (44.0–310.0)	18.889	82.167	−13.615	51.393	0.243
LDL (mg/dL)	88.1 ± 22.5 (48.0–144.0)	81.2 ± 19.9 (49.0–144.0)	7.130	29.910	−3.842	18.100	0.194
HDL (mg/dL)	50.3 ± 11.8 (28.0–77.0)	52.5 ± 12.9 (27.0–83.0)	−2.115	5.701	−4.418	0.187	0.070

**Table 3:** Multivariable linear regression analysis for factors associated with CMRI.

	Δ Myocardial TG		Δ Pericardial fat		Δ ECV	
	Standardize d coefficient β	p-Value	Standardize d coefficient β	*p*	Standardize d coefficient β	p-Value
**Difference CMRI**						
Δ ECV			0.802	**0**.**028**		
Δ LVMi					0.075	**0**.**020**
**Baseline CMRI**						
Myocardial TG	−0.806	<**0**.**001**				
Pericardial fat			−0.834	<**0**.**001**		
ECV					−0.710	<**0**.**001**
LVEF					−0.172	<**0**.**001**

Δ: Difference after empagliflozin treatment.

## Discussion

In this study, we showed that, overall, 6 months of empagliflozin treatment did not significantly improve the LV function, structure, adiposity, and diffuse fibrosis in patients with T2DM. We also demonstrated that the beneficial effects of empagliflozin treatment might be more evident in patients with worse baseline LV substrate and structure.

T2DM is an independent risk factor of heart failure with adverse effects on the function and structure of the heart^15^. The development of diabetic cardiomyopathy is slow and silent when there are no other confounding factors such as CAD, hypertension, and valvular heart disease. Diastolic dysfunction is considered the most frequent and an early sign of myocardial dysfunction that could emerge even in the early stage of T2DM^16–18^. The pathogenesis of diabetic cardiomyopathy is complex. In normal individuals, myocardial energy is mainly derived from the metabolism of glucose and free fatty acids through the lipolysis of adipose tissue; however, in diabetic patients, the energy sources are shifted to the beta-oxidation of fatty acids because of the depletion of glucose transporters 1 and 4, in which the oxygen demand is higher and energy imbalance occurs. Free fatty acids inhibit pyruvate dehydrogenase, accumulate glycolytic intermediates and ceramide, and further promote apoptosis^15,19^. In a previous study using 1 H magnetic resonance spectroscopy (MRS), myocardial TG content was increased in diabetic patients and associated with impaired LV diastolic function^14,20^. Although the proportion of patients with systolic versus diastolic dysfunction in the EMPA-REG OUTCOME trial is unknown, diastolic dysfunction was reported to be present in >60% of subjects with diabetes^21^.

Cardiac MRI has provided a unique view of subclinical lipid overload-related myocardial remodelling. Studies using cardiac MRI have established an association between myocardial steatosis and LV dysfunction independent of age, diastolic blood pressure, and BMI in T2DM^14,22,23^. However, the effects of therapeutic interventions on the myocardial substrate of patients with T2DM have been seldom studied. One report showed that 16 weeks of caloric restriction in patients with T2DM seemed to decrease the myocardial TG content along with improvements in LV diastolic function, LV mass, and E/A ratio^24^. Another study showed that although pioglitazone, in comparison with metformin, could improve LV function and alter the myocardial substrate metabolism in patients with T2DM, no therapeutic effect on myocardial TG content was noted^25^. Empagliflozin is the first oral anti-hyperglycaemic agent proven to have benefits on the CV outcome. Therefore, it is of clinical interest to determine if empagliflozin would have beneficial effects on the heart function/structure and myocardial substrate. In one human study, 3 months of empagliflozin treatment was associated with a significant reduction in LVMi and an improvement in diastolic function^8^. Recently, Verma *et al*. conducted the EMPA-HEART CardioLink-6 trial and enrolled 97 patients with T2DM and established CAD. This study showed that 6 months of empagliflozin treatment could lower the LVMi and that, when LVMi was >60 g/m^2^, the regression of LV mass was associated with the baseline LV mass^26^.

Despite the impressive results, the potential mechanisms remain largely unexplained. The unique pharmacological profile of SGLT2 inhibitors places them at the crossroads of important haemodynamic, neurohumoral, metabolic, and vascular endothelial pathways influencing cardiac and renal diseases. There are several potential direct and indirect pathways leading to the improvement of heart structure, function, and myocardial substrate induced by SGLT2. Treatment with SGLT2 inhibitors in patients with diabetes could provide better glycaemic control and insulin sensitivity and reduce arterial blood pressure, arterial stiffness, body weight, and visceral fat^27–30^. Recent studies further demonstrated that SGLT2 inhibitors could reset the tubuloglomerular feedback, leading to volume contraction without the activation of the sympathetic nervous system and harmful glomerular hyperfiltration^31^. Moreover, SGLT2 inhibitors might improve the efficiency of myocardial energy utilization by offering β-hydroxybutyrate as an attractive fuel for oxidation^6^. These multiple non-glycaemic effects support SGLT2 inhibitors as the preferred glucose-lowering drug for treating diabetic patients with heart failure.

Although the current study failed to demonstrate improvements in heart function/structure and myocardial substrate, we found that the greater the accumulation of intracardiac TG content, pericardial fat, and diffuse fibrosis at baseline, the better the improvements of these components after 6 months of empagliflozin treatment. The discrepancy between our findings and those of previous studies^8,26^ might be attributed to the different study populations and the lower severity of T2DM, fewer comorbidities, normal or milder heart function impairment, and myocardial substrate change in our study. Nevertheless, our results are in accordance with the current observations that patients with worse baseline characteristics benefit more from the treatment.

The adiposity of the heart has been proven to be significantly associated with heart function. For example, pericardial fat is associated with metabolic risks, severity of CAD, adverse LV remodelling, and sudden cardiac death^32–35^. The impact of SGLT2 inhibitors on the adiposity and fibrosis content of the liver has been addressed by several studies. For example, tofogliflozin and ipragliflozin have been shown to prevent hepatic TG accumulation and fibrosis in obese rats^36^. A study pooling randomized trials including EMPA-REG demonstrated a highly consistent result that empagliflozin reduces aminotransferases in patients with T2DM, which potentially indicates a reduction in liver fat^30^. Thus far, there is no report on the effects of SGLT2 on the adiposity (cardiac steatosis and pericardial fat) and fibrosis content of the heart. As the first study enrolling patients with worse baseline disease severity and cardiac characteristics, our current study might be important in clarifying these issues.

This study has some limitations. First, the study population was small and no control (non-diabetic) group was enrolled in this study. Second, instead of echocardiographic measurements, we used only LVPFR to represent LV diastolic function, which might be an insensitive indicator. Third, we used non-invasive MRS to quantify the intracardiac TG content and myocardial diffuse fibrosis without performing histological examination to validate the results. Fourth, whether the results of this study on empagliflozin can be extrapolated to all SGLT2 inhibitors is questionable. Finally, the lack of a significant reduction of HbA1c may raise the possibility that some patients might have poor compliance with the medication despite our careful efforts to confirm the compliance of patients. Nevertheless, the significant increase in haematocrit, reduction in blood pressure, and persistent glucosuria during the study imply that most of the patients had fair compliance.

In conclusion, we demonstrated that, overall, 6 months of empagliflozin treatment did not significantly improve the LV function, structure, adiposity, and diffuse fibrosis in patients with T2DM. However, we found that the beneficial effects of empagliflozin on the intracardiac TG content, pericardial fat, and ECV might be more evident in patients with worse baseline LV substrate and structure.

## Methods

### Study design and population

The study was approved by the institutional review board of the National Taiwan University Hospital Ethics Committee. All participants provided written informed consent. We prospectively enrolled a total of 41 patients with T2DM aged >20 years from June 1, 2017, to November 31, 2018. The patients received an SGLT2 inhibitor (empagliflozin 25 or 12.5 mg/d) for 6 months in addition to a stable regimen of oral hypoglycaemic agents. The diagnosis of T2DM was based on the 2018 American Diabetes Association criteria, as follows: fasting plasma glucose ≥126 mg/dL (7.0 mmol/L), or 2-h plasma glucose ≥200 mg/dL (11.1 mmol/L) during an oral glucose tolerance test, or A1C ≥6.5% (48 mmol/mol), or random plasma glucose ≥200 mg/dL (11.1 mmol/L) in a patient with classic symptoms of hyperglycaemia or hyperglycaemic crisis. Detailed medical history was obtained including comorbid diseases and current medications. The exclusion criteria were chronic kidney disease stage V or end-stage renal disease that precluded the treatment of empagliflozin or injection of gadolinium. As LV function might be influenced by CAD during the study, patients with significant CAD with active ischaemia or those who had undergone a coronary intervention procedure within 6 months before enrolment were also excluded. The clinical data, body weight, systolic blood pressure, diastolic blood pressure, and laboratory information including total cholesterol, TG, low-density lipoprotein, high-density lipoprotein, HbA1c, and NT-proBNP were all recorded before and after empagliflozin treatment. All enrolled patients underwent CMRI before and after empagliflozin therapy. Among them, four patients were excluded because of poor CMRI quality or incomplete data and two patients withdrew from the study because of intolerance to empagliflozin.

### Cardiac magnetic resonance image acquisition

A 1.5-T MRI system (Trio: Siemens, Erlangen, Germany) with an eight-channel CV phased array torso coil was used. The Modified Look Locker Inversion recovery (MOLLI) sequence for myocardial T1 mapping was performed in a single breath hold before and 10 min after the intravenous administration of 0.15 mmol/kg gadolinium-based contrast medium (Omniscan; Winthrop Laboratories, GE, Princeton, NJ, USA). During three to five heartbeats after the inversion pulse, the electrocardiography (ECG)-triggered MOLLI protocol obtained images intermittently in the diastole phases, generating each inversion recovery curves for the region of interest (ROI) and each pixel location. The information of multiple inversion recovery of each pixel location can be applied to generate T1 mapping^37^.

Cine MRI was performed with an ECG-triggered segmented, balanced, steady-state gradient echo pulse sequence. Short-axis slices were separated from the mitral orifice to the LV apex with a slice thickness of 8 mm and a gap of 2 mm. The T1 maps were reconstructed using the scanner console. We adjusted the centre frequency to avoid off-resonance artefacts and excluded image with poor quality due to severe motion artefacts secondary to arrhythmia or difficulty in breath holding.

Cardiac MRS was performed by using a standard flex coil for signal reception. Four-chamber and short-axis cine images were used to prescribe the locations of MRS voxels. The H1-magnetic resonance spectra of water and TG were acquired using cardiac-gated point-resolved spectroscopy during a single breath hold. The acquisition parameters were as follows: nominal repetition time/echo time, 550 ms/30 ms; 10–12 average; and voxel size, 4*23*20 mm^3^. The myocardial TG signal was acquired at 1.4 ppm from the spectra with water suppression, and the water signal was acquired at 4.7 ppm from the spectra without water suppression^38^.

### Cardiac magnetic resonance image analysis

The main MRI parameters in this study were ECV, LVEDV, LVESV, LVEF, LVPER, LVPFR, and LV mass at end-diastolic volume, LV intracardiac fat (TG) content, and pericardial fat.

The definitions were as follows:

- ECV: The quantitative analysis of myocardial ECV was based on T1 maps. For each slice, the ROIs in blood and in the LV myocardium were drawn in the central area of the LV cavity and the septal myocardium. The T1 values of the segmented ROIs were averaged. The difference between the pre-contrast value and the post-contrast value represented the changes of the relaxation rate (1/T1). The ratio of the change in relaxation rate (1/T1) in the myocardium to that in blood, adjusted by multiplying by (1-haematocrit), is defined as ECV.
- LV function and structure: The parameters were obtained from cine MRI. For each slice level, the endocardial and epicardial contours of the left ventricle were determined. Using Simpson’s rule for the volume-time curve of the left ventricle, the LV volumes for each heart cycle were calculated. LV end-systolic diameter and LVESV were defined as the maximal and minimal LV values in the volume-time curve. LV mass was defined as the difference between LV epicardial volume at end-diastole and LVEDV, multiplied by the myocardial density, which is 1.05 g/cm^3^. All indices of LV volumes and mass were normalized by dividing with the body surface area.
- Pericardial fat: Consecutive short-axis images during the end-diastolic phase were examined and multiplied by the slice thickness. Pericardial fat was defined as regions of high-signal intensity between the epicardium and the endocardium.
- Intracardiac fat: In the magnetic resonance spectrum, signal suppression with hydrogen was manipulated, and there were two peaks representing the water and TG proportions, respectively. The myocardial TG content was expressed as the integral ratio of TG to water (%).

All parameters were analysed using a custom-developed MATLAB 7.9 program (MathWorks, Natick, MA, USA).

### Statistics

Continuous variables are presented as means ± standard deviations, and categorical variables are presented as numbers and percentages. The differences of clinical, laboratory, and MRI parameters before and after the treatment with empagliflozin were analysed using paired Student’s t-test. To investigate the determinants of the changes of MRI parameters before and after empagliflozin treatment, stepwise multiple linear regression analysis was performed using changes of the cardiac TG content, pericardial fat, or ECV as dependent variables and the baseline values or changes of other MRI parameters including normalized LV end-diastolic diameter, normalized LV end-systolic diameter, LVMi, LVEF, LVPER, and LVPFR as independent variables. Two-tailed p < 0.05 was considered statistically significant. All analyses were conducted using Statistical Package for the Social Sciences software (version 25.0; SPSS Inc., Chicago, IL, USA).

### Ethical approval

The study was approved by the institutional review board of the National Taiwan University Hospital Ethics Committee.

### Informed consent

Written consent was collected from all patients. The study complied with the second Declaration of Helsinki. The design of the study was approved by a regional scientific ethics committee.

## Data Availability

The datasets generated during and/or analysed during the current study are available from the corresponding author on reasonable request.
